# Disseminated melioidosis complicated by symmetrical peripheral gangrene and multi-organ failure: a case report

**DOI:** 10.3389/fimmu.2026.1840124

**Published:** 2026-07-21

**Authors:** Chen-Hsuan Lin, Ping-Chang Lin, Ching-Yi Tsai, Jih-Jin Tsai

**Affiliations:** 1 School of Medicine, College of Medicine, Kaohsiung Medical University, Kaohsiung, Taiwan; 2 Tropical Medicine Center, Kaohsiung Medical University Hospital, Kaohsiung, Taiwan; 3 Division of Infectious Diseases, Department of Internal Medicine, Kaohsiung Medical University Hospital, Kaohsiung, Taiwan

**Keywords:** *Burkholderia pseudomallei*, melioidosis, prostatic abscess, septic shock, symmetrical peripheral gangrene

## Abstract

**Background:**

Melioidosis, caused by *Burkholderia pseudomallei*, is a potentially fatal infection endemic to Southeast Asia and northern Australia. Disseminated disease with bacteremia and multifocal organ involvement carries high mortality, particularly among patients with diabetes mellitus (DM) and hazardous alcohol use. Symmetrical peripheral gangrene (SPG) is a rare complication that may result in permanent disability.

**Case presentation:**

A 57-year-old man with poorly controlled type 2 DM, chronic alcohol use, and malnutrition presented with septic shock and acute hypoxemic respiratory failure. Imaging revealed cavitary pneumonia and abscesses involving the liver, kidney, and prostate. Two independently collected blood cultures and a prostatic abscess aspirate yielded *B. pseudomallei*, confirmed through a multistep microbiological workflow with final VITEK 2 identification. The patient required vasopressors, mechanical ventilation, continuous venovenous hemofiltration, intravenous meropenem, and image-guided drainage of the prostatic abscess. Distal cyanosis was present at admission and progressed to dry gangrene of multiple fingers and toes. The symmetric acral distribution and absence of major-vessel obstruction were clinically consistent with SPG. Recurrent hyperkalemia and renal dysfunction prevented continued trimethoprim-sulfamethoxazole therapy, necessitating six months of doxycycline plus amoxicillin-clavulanate. Secondary infection and osteomyelitis of the gangrenous digits required multiple amputations. At follow-up 11 months after completion of eradication therapy, there was no evidence of recurrent melioidosis.

**Conclusion:**

SPG is a rare but devastating complication of disseminated melioidosis. Prompt antimicrobial therapy, intensive organ support, source control, and early recognition of peripheral ischemia are important. Even after survival and successful treatment, substantial long-term functional disability may persist.

## Introduction

Melioidosis is a potentially fatal infection caused by *Burkholderia pseudomallei*, a Gram-negative, facultative intracellular bacillus endemic to Southeast Asia and northern Australia (1). In Taiwan, cases occur predominantly in southern coastal regions, with incidence increasing after heavy rainfall and typhoons, likely because of environmental exposure to contaminated soil and surface water (2). Infection occurs through percutaneous inoculation, inhalation, or ingestion (3). The clinical spectrum of melioidosis is notably broad, ranging from localized skin and soft-tissue infection to fulminant septic shock with multi-organ failure (4). This heterogeneity, coupled with clinical features that overlap with tuberculosis, malignancy, and other severe bacterial infections, frequently delays diagnosis and initiation of appropriate therapy (5).

Disseminated melioidosis represents the most severe form of the disease and is characterized by bacteremia with multifocal involvement, commonly affecting the lungs, liver, spleen, kidneys, and genitourinary tract (6). Among male patients, prostatic abscesses are increasingly recognized as a clinically significant yet frequently underdiagnosed focus of infection, with implications for persistent bacteremia and disease relapse if not promptly recognized (7). Despite advances in antimicrobial therapy and critical care, disseminated disease remains associated with substantial mortality, particularly among patients presenting with septic shock (4).

Host factors play a central role in disease susceptibility and severity. Diabetes mellitus (DM) is the strongest and most consistently reported risk factor for melioidosis and confers up to a twelve-fold increased risk of infection (6). Other recognized risk factors include hazardous alcohol use, chronic kidney disease, and prolonged corticosteroid therapy, all of which impair innate immune function and facilitate intracellular persistence of *B. pseudomallei* (6). In endemic regions, geographic and occupational exposures further modulate infection risk.

Although melioidosis is well known for its propensity for multifocal dissemination, severe peripheral ischemic complications are exceedingly rare, poorly characterized, and the underlying mechanism is still incompletely understood.

We present a case of disseminated melioidosis complicated by symmetrical peripheral gangrene (SPG) and multi-organ failure, highlighting the diagnostic and therapeutic challenges, the uncertain mechanism of peripheral ischemia, and the substantial long-term functional morbidity associated with severe disease in an endemic setting.

## Case description

A 57-year-old man presented with five days of progressive generalized weakness, unsteady gait, worsening dyspnea, chronic dry cough, and urinary frequency without dysuria. During the preceding two months, he had poor appetite and significant unintentional weight loss. His medical history was notable for poorly controlled type 2 DM, chronic alcohol use, and benign prostatic hyperplasia. He lived and worked in a rural area with frequent exposure to soil and surface water. His clinical course is summarized in **Table 1**.

**Table 1:** Key clinical events from presentation to long-term follow-up.

Time point	Key events
2 months before admission	Progressive poor appetite and significant unintentional weight loss
5 days before admission	Onset of generalized weakness, unsteady gait, worsening dyspnea, chronic dry cough, and urinary frequency without dysuria
Day 0	Emergency department presentation with hypotension, tachycardia, hypoxemia, and cool distal extremities with digital cyanosis; labs notable for lactic acidosis, leukocytopenia with a left shift, thrombocytopenia, acute kidney injury, and transaminitis; blood cultures collected
Days 0–1	ICU admission for septic shock and acute hypoxemic respiratory failure; empiric ceftriaxone and levofloxacin initiated for severe community-acquired pneumonia
Day 1	Rapid deterioration with multi-organ failure; antimicrobials escalated to ertapenem; initiation of vasopressors, mechanical ventilation, and CVVH
Day 2	Meropenem initiated because of continued clinical deterioration; teicoplanin added for empiric Gram-positive coverage
Day 4	Blood cultures collected at admission were reported positive for *Burkholderia pseudomallei*, confirming disseminated melioidosis; AFB studies negative; meropenem continued and TMP-SMX initiated
Day 6	CVVH discontinued following improvement in renal function and metabolic status
Day 7	Vasopressors discontinued; digital cyanosis present since admission had progressively worsened and subsequently evolved into dry gangrene
Day 9	Image-guided drainage of the prostatic abscess
Day 12	Prostatic abscess aspirate reported positive for *B. pseudomallei*; initial TMP-SMX course discontinued because of hyperkalemia
Day 14	Meropenem temporarily replaced by cefoperazone-sulbactam and inhaled colistin because of concern for a carbapenem-resistant nosocomial pathogen
Day 20	Mechanical ventilation discontinued
Day 22	Transferred to the infectious diseases ward; intravenous meropenem resumed for continued treatment through discharge
Day 39	TMP-SMX rechallenged but discontinued after 3 days due to recurrent hyperkalemia and worsening renal function
Day 59	Discharged after 59 days of hospitalization; approximately 48 cumulative days of intravenous meropenem across two treatment periods; transitioned to oral eradication therapy with doxycycline and amoxicillin-clavulanate
3 months after discharge	Right index finger swelling and purulent discharge; cultures grew *Pseudomonas aeruginosa* followed by MRSA; bone scintigraphy confirmed osteomyelitis involving the right second digit
4 months after discharge	First surgery: amputation of the right index finger (to the middle phalanx) and the left third and fourth fingers
6 months after discharge	Completion of oral eradication therapy; bilateral toe gangrene progressed to spontaneous auto-amputation
7 months after discharge	Second surgery: amputation/sequestrectomy of the right third and fourth fingers with advancement flap
11 months after completion of eradication therapy	No evidence of recurrent melioidosis; ongoing rehabilitation and counseling regarding strict glycemic control and alcohol cessation

AFB, acid-fast bacilli; CVVH, continuous venovenous hemofiltration; ICU, intensive care unit; MRSA, methicillin-resistant *Staphylococcus aureus*; TMP-SMX, trimethoprim-sulfamethoxazole.

On arrival, he appeared acutely ill and drowsy, with hypotension (96/66 mmHg), tachycardia (137 beats/min), and hypoxemia (SpO_2_ 90% despite oxygen through a non-rebreather mask at 12 L/min). His body mass index was 16.8 kg/m^2^. Examination showed respiratory distress with accessory muscle use and crackles over the right lung field. Distal extremities were cool with cyanotic discoloration but no tissue necrosis.

Initial laboratory evaluation demonstrated poor glycemic control (HbA1c 9%), leukocytopenia witha left shift (white blood cell count 1,050/μL; band forms 8%), thrombocytopenia (35,000/μL), acute kidney injury (creatinine 2.29 mg/dL), elevated lactate (4.33 mmol/L), C-reactive protein > 160 mg/L, transaminitis (aspartate aminotransferase 615 IU/L, alanine aminotransferase 144 IU/L), and hypoalbuminemia (2.08 g/dL). Coagulation studies showed a prothrombin time (PT) of 15.1 seconds, international normalized ratio (INR) of 1.57, and activated partial thromboplastin time of 45.3 seconds. Subsequent testing showed an elevated D-dimer level of 4.53 mg/L fibrinogen-equivalent units, while protein C and protein S activities were within normal ranges at 104% and 82%, respectively. Key laboratory results are presented in **Supplementary Table 1**.

**Figure 1:**
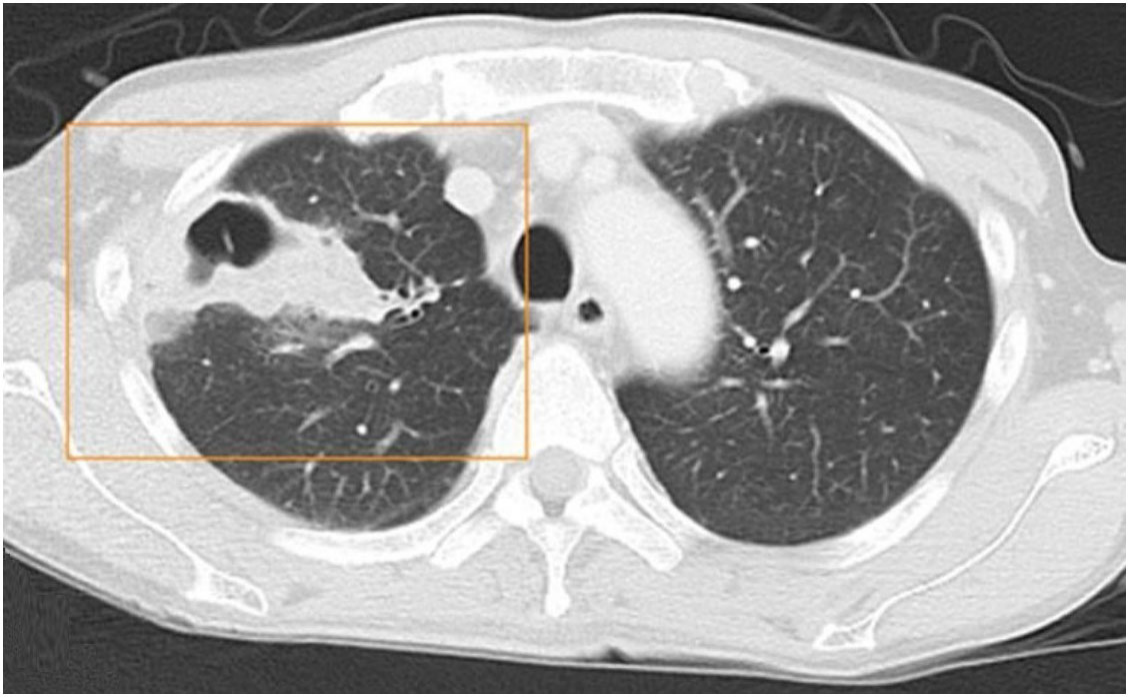
Chest computed tomography showed a 2.6-cm cavitary lesion in the right upper lobe with bilateral pulmonary infiltrates.

**Figure 2:**
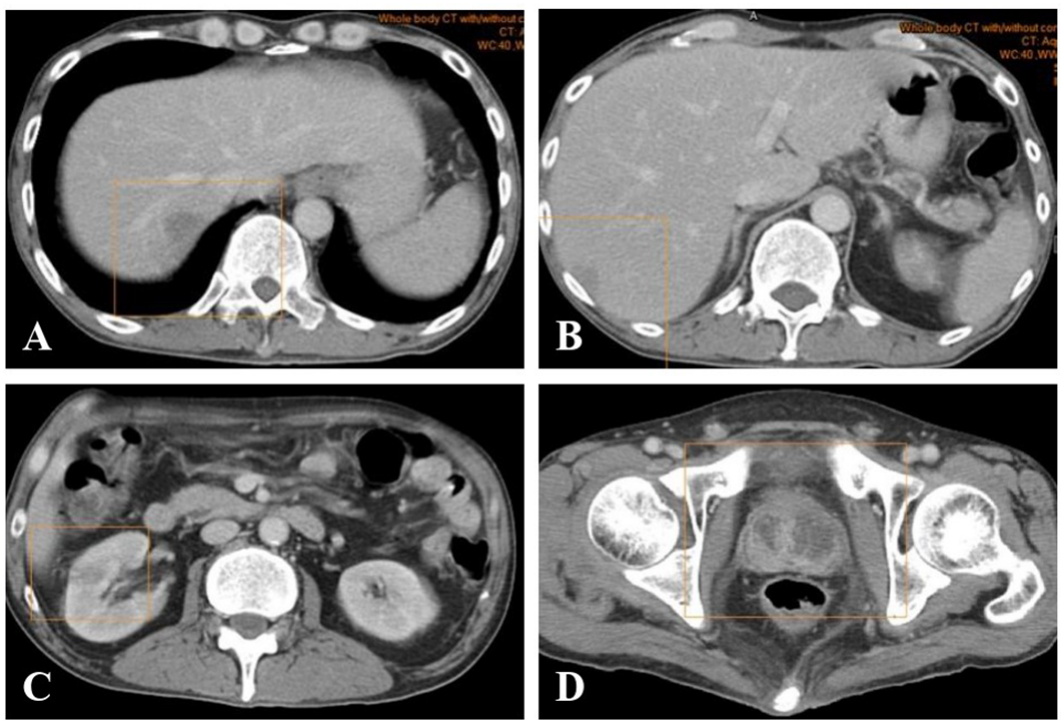
Whole-body computed tomography revealed **(A, B)** poorly enhanced hypodense lesions in segment VII of the liver, consistent with hepatic abscesses; **(C)** a 1.5-cm abscess in the right kidney; and **(D)** a hypodense lesion within the prostate, later confirmed as a prostatic abscess on drainage.

Chest imaging revealed a 2.6-cm cavitary lesion in the right upper lobe with bilateral pulmonary infiltrates (**Figure 1**). Whole-body computed tomography further identified hypodense lesions suspicious for abscesses in the liver, right kidney, and prostate (**Figure 2**).

The patient was admitted to the medical intensive care unit (MICU) with septic shock and acute hypoxemic respiratory failure. Ceftriaxone and levofloxacin were initiated empirically for severe community-acquired pneumonia. Following rapid deterioration with multi-organ failure, treatment was escalated to ertapenem on hospital day 1 and meropenem on day 2, with teicoplanin added for empiric Gram-positive coverage. He required endotracheal intubation, mechanical ventilation, and continuous venovenous hemofiltration (CVVH) for refractory metabolic acidosis and oliguria. On day 1, vasopressin was started at 0.03 units/min, briefly increased to 0.05 units/min, and discontinued within 24 hours. Norepinephrine was started at 5.17 µg/min, peaked at 12.92 µg/min, and was tapered off by day 7.

Two blood-culture specimens collected at admission were reported positive for *B. pseudomallei* on day 4. Suspect isolates were recognized by characteristic colony morphology and bipolar Gram staining, with adjunctive matrix-assisted laser desorption/ionization time-of-flight (MALDI-TOF) mass spectrometry and final identification by VITEK 2 in a Taiwan Centers for Disease Control-authorized laboratory. Antimicrobial susceptibility testing, interpreted using historical Clinical and Laboratory Standards Institute (CLSI)-based criteria, showed susceptibility to trimethoprim-sulfamethoxazole (TMP-SMX), ceftazidime, and imipenem, supporting continued meropenem and the addition of TMP-SMX on day 4. CVVH was discontinued on day 6 with gradual hemodynamic and renal improvement.

Image-guided drainage of the prostatic abscess was performed on day 9. The aspirate came back positive for *B. pseudomallei* on day 12 and showed the same susceptibility profile as the blood isolates. The initial intravenous TMP-SMX course was dose-adjusted but discontinued after eight days because of hyperkalemia. Meropenem was temporarily replaced on day 14 because of concern for a carbapenem-resistant nosocomial pathogen. Mechanical ventilation was discontinued on day 20. On day 22, the patient was transferred to the infectious diseases ward, where meropenem was resumed and continued through discharge.

Digital cyanosis progressively worsened during the first hospital week and evolved into dry gangrene of multiple fingers and toes. Transthoracic echocardiography showed no valvular vegetations, while vascular imaging demonstrated no large-vessel occlusion or peripheral arterial disease. Testing for autoimmune and vasculitic disorders, including a connective tissue disease screening panel, anticardiolipin antibodies, anti-β2-glycoprotein I IgG, proteinase 3- and myeloperoxidase-antineutrophil cytoplasmic antibodies, and an extended myositis panel, was unremarkable. The symmetric acral distribution and absence of major-vessel obstruction were clinically consistent with SPG.

Oral TMP-SMX was rechallenged on day 39 but discontinued after three days because of recurrent hyperkalemia and worsening renal function. The patient was discharged on day 59 after approximately 48 cumulative days of intravenous meropenem. Because TMP-SMX could not be tolerated, he was transitioned to doxycycline plus amoxicillin-clavulanate for six months of eradication therapy.

**Figure 3:**
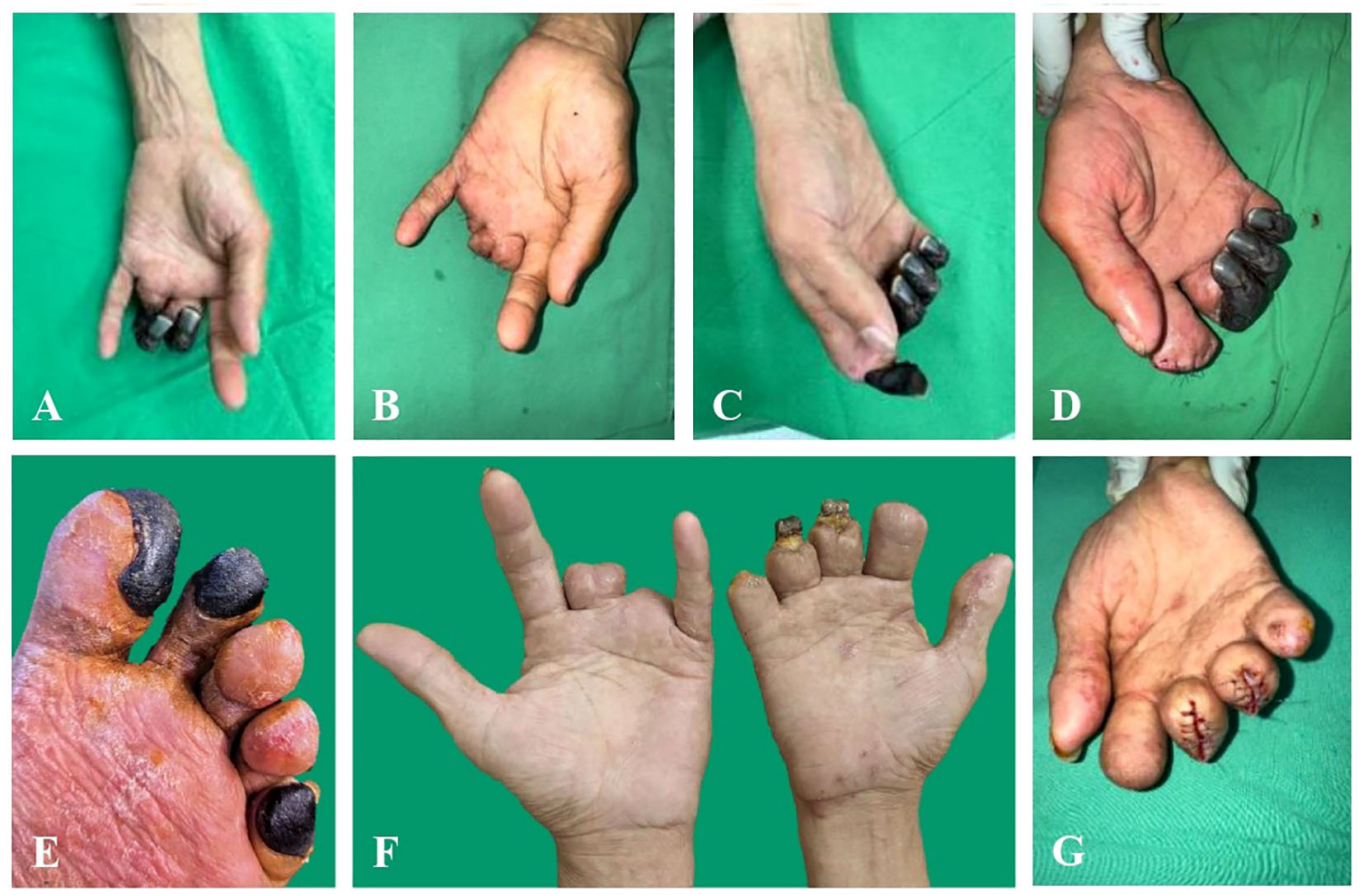
Digital ischemia and surgical management of gangrene in chronological order. **(A)** Left hand before first operation. **(B)** Left hand after first operation: amputation of the third and fourth fingers. **(C)** Right hand before first operation. **(D)** Right hand after first operation: amputation of the index finger at the middle phalanx. **(E)** Toe gangrene progressed to spontaneous auto-amputation without surgical intervention. **(F)** Both hands photographed at follow-up. **(G)** Right hand after second operation: amputation of the third and fourth fingers.

Three months after discharge, the gangrenous right index finger developed swelling and purulent discharge. Wound cultures grew *Pseudomonas aeruginosa* and subsequently methicillin-resistant *Staphylococcus aureus* (MRSA). Bone scintigraphy suggested osteomyelitis of the right second digit. Four months after discharge, the right index finger was amputated to the middle phalanx together with the left third and fourth fingers (**Figure 3**). Histopathologic examination demonstrated gangrenous necrosis with suppurative inflammation involving the skin, soft tissue, and bone. Three months later, persistent infection in nonviable tissue required amputation and sequestrectomy of the right third and fourth fingers; histopathology confirmed necrotic bone with sequestrum and osteomyelitis. Bilateral toe gangrene progressed to spontaneous auto-amputation.

At follow-up approximately 17 months after discharge and 11 months after completion of eradication therapy, there was no evidence of recurrent melioidosis. The patient continued rehabilitation and received repeated counseling regarding glycemic control and alcohol cessation.

## Discussion

This case illustrates the protean and destructive potential of disseminated melioidosis in a susceptible host. The patient presented with bacteremia, cavitary pneumonia, multiple visceral abscesses, septic shock, and multi-organ failure, followed by SPG with permanent functional disability. Although visceral dissemination is well recognized in melioidosis (6), extensive acral ischemia affecting both the fingers and toes is unusual and suggests that survival from fulminant infection may be accompanied by substantial long-term morbidity.

The pulmonary, hepatic, renal, and prostatic involvement reflected widespread hematogenous dissemination. Prostatic abscesses are an important but potentially underrecognized focus in men with melioidosis and may contribute to persistent infection if not identified and drained (7). In this patient, urinary frequency prompted evaluation of the genitourinary tract, and image-guided drainage provided source control. Poorly controlled type 2 DM and chronic alcohol use likely increased both susceptibility and disease severity through impaired host immune responses (4, 7, 8).

Microbiological identification of *B. pseudomallei* requires caution because automated systems, including VITEK 2, have occasionally misidentified the organism as *B. cepacia* complex or other Gram-negative bacilli (9, 10). In this case, diagnostic confidence was strengthened by the multistep laboratory workflow and concordant identification from two independently collected blood cultures and a separately obtained prostatic abscess aspirate, together with consistent susceptibility profiles and a compatible clinical and radiological presentation in an endemic region. Molecular confirmation was not performed and remains a limitation.

Several factors likely contributed to survival despite fulminant disseminated disease. Meropenem was initiated on day 2 during rapid clinical deterioration, before the final culture identification became available on day 4. Early MICU admission allowed prompt hemodynamic, respiratory, and renal support with vasopressors, mechanical ventilation, and CVVH. Identification and drainage provided rapid source control for the prostatic infection, and familiarity with melioidosis in southern Taiwan may have facilitated recognition once *B. pseudomallei* was reported.

Peripheral ischemic complications have rarely been reported in melioidosis. One previous case considered high inotropic support and possible septic embolization as causes of digital gangrene (11), whereas another described toe gangrene in recurrent disease following incomplete eradication therapy (12). In this patient, cyanosis was present at admission and progressed to SPG involving both the fingers and toes without major-vessel obstruction. SPG is associated with shock-related microvascular thrombosis and disseminated intravascular coagulation (DIC), often accompanied by depletion of natural anticoagulants such as protein C and antithrombin (13).

Severe thrombocytopenia, prolonged PT/INR, markedly elevated D-dimer, and multi-organ dysfunction were consistent with sepsis-induced coagulopathy and compatible with overt DIC (14). Based on the available International Society on Thrombosis and Haemostasis (ISTH) overt-DIC score components, our patient scored six points: two points for a platelet count below 50,000/µL, one point for PT prolongation exceeding three seconds, and three points for a strongly elevated D-dimer; fibrinogen was unavailable. A score of more than five points indicates overt DIC. Protein C and protein S activities were normal, and antithrombin was not measured, so natural anticoagulant depletion was not demonstrated. Histopathology obtained approximately 6 and 9 months after ischemia onset showed gangrenous necrosis, suppurative inflammation, sequestrum, and osteomyelitis. No microvascular thrombi were reported; however, an earlier thrombotic process could have led to rapid gangrene.

The onset of cyanosis before vasopressor administration argues against vasopressors as the initiating cause, although vasoconstriction may have aggravated the ischemia (15). Heparin-induced thrombocytopenia was also unlikely because thrombocytopenia and cyanosis were present before any potential heparin exposure, although specific testing was not performed. Overall, sepsis-associated DIC with microvascular thrombosis was considered the most plausible mechanism.

Treatment of disseminated melioidosis requires an intensive intravenous phase followed by at least three months of oral eradication therapy (16). Meropenem is appropriate for critically ill patients, while TMP-SMX is added when genitourinary or other non-pulmonary focal infection is present (4, 16). Current guidance recommends graded TMP-SMX initiation and folic acid supplementation to reduce antifolate-related hematologic toxicity (17). In this case, TMP-SMX was dose-adjusted but not introduced using a formal graded protocol. Recurrent hyperkalemia and worsening renal function nevertheless prevented continued therapy despite dose modification.

Because TMP-SMX could not be tolerated, doxycycline plus amoxicillin-clavulanate was selected as an alternative eradication regimen. Clinical evidence for this combination is limited to small case series and case reports; however, all reported patients remained recurrence-free during the available follow-up (18–20). Microbiological studies provide indirect support, showing that most *B. pseudomallei* isolates remain susceptible to both agents and that their combination does not exhibit *in vitro* antagonism, although synergy has not been demonstrated (21, 22). Thus, the regimen is biologically plausible and supported by limited clinical experience, but its efficacy relative to TMP-SMX remains uncertain.

The osteomyelitis developed in previously ischemic digits and was associated with secondary *P. aeruginosa* and MRSA infection; however, a contribution from *B. pseudomallei* could not be excluded. To mitigate the risk of relapse in melioidosis-associated bone involvement, a six-month course of eradication therapy was initiated, consistent with guideline recommendations (17). Prolonged critical illness, antimicrobial exposure, organ dysfunction, and nonviable tissue likely predisposed the gangrenous digits to superinfection and subsequent amputation (23). This presentation differed from primary musculoskeletal melioidosis, which more commonly manifests as septic arthritis or hematogenous osteomyelitis (24).

The geographic context remains clinically relevant. Southern Taiwan is an endemic but potentially underrecognized setting, and this patient had frequent rural exposure to soil and surface water (25, 26). Melioidosis should therefore be considered in patients with severe community-acquired sepsis, cavitary pneumonia, or multifocal visceral abscesses, particularly when DM or hazardous alcohol use is present. This case also demonstrates that microbiological eradication and survival do not preclude major long-term disability. Close follow-up, rehabilitation, and modification of relapse-associated risk factors, including poor glycemic control and alcohol use, remain essential components of recovery.

## Take-away lessons

Disseminated melioidosis may be complicated by SPG, with peripheral ischemia developing before vasopressor exposure and no identifiable embolic source. Early recognition, prompt initiation of appropriate antimicrobial therapy, and source control of occult foci are essential for survival. However, as demonstrated in this case, successful microbiological eradication does not preclude significant long-term morbidity. Monitoring of peripheral ischemia, along with risk-factor modification, is critical to optimizing functional outcomes in high-risk patients.

## Patient perspective

I went to the hospital because I felt extremely weak, short of breath, and unsteady when walking. At first, I thought it was just exhaustion, but my condition worsened very quickly. I was later told that I had a serious infection and had to stay in the ICU. At one point, my family signed a do-not-resuscitate order due to my severe condition. During that time, I was very sick and often confused, and I do not remember much of it except being connected to many machines that helped keep me alive. After leaving the hospital, I also needed to continue taking antibiotics for a long period of time.

Although I gradually recovered from the infection, I began to notice worsening discoloration and pain in my fingers and toes. Learning that the circulation in my fingers could not recover and that amputation was necessary was emotionally difficult. After the surgery, I needed physical therapy to relearn daily activities and regain strength. Although surviving the illness was a relief, losing part of my fingers has had a lasting impact on my daily life. Ongoing rehabilitation has been important in helping me adjust physically and emotionally.

## Data Availability

The original contributions presented in the study are included in the article/**Supplementary Material**. Further inquiries can be directed to the corresponding author.

## Supplementary material

Supplementary Table 1 Key laboratory findings during admission.
