# Supplementary material for: Disseminated melioidosis complicated by symmetrical peripheral gangrene and multi-organ failure: a case report
Source: Front Immunol. 2026 Jul 21;17:1840124. doi: 10.3389/fimmu.2026.1840124 (PMC13433207; doi:10.3389/fimmu.2026.1840124)
Supplement: Supplementary file 1 [file Table1.docx]

**Supplementary Table 1. Key laboratory findings during admission.**

| Category | Laboratory parameter | Result | Institutional reference range |
| --- | --- | --- | --- |
| Glycemic status | HbA1c | 9.0% | 4–6% |
| Hematologic | WBC count | 1,050/µL | 3,920–9,240/µL |
|  | Band forms | 8% | 0–5% |
|  | Platelet count | 35,000/µL | 171,000–400,000/µL |
| Renal/metabolic | Creatinine | 2.29 mg/dL | 0.60–1.20 mg/dL |
|  | Lactate | 4.33 mmol/L | 0.5–2.2 mmol/L |
| Inflammatory | CRP | > 160 mg/L | ≤ 5 mg/L |
| Hepatic/nutritional | AST | 615 IU/L | 11–34 IU/L |
|  | ALT | 144 IU/L | < 45 IU/L |
|  | Albumin | 2.08 g/dL | 3.5–5.0 g/dL |
| Coagulation | PT | 15.1 seconds | 8–12 seconds |
|  | INR | 1.57 | 0.85–1.15 |
|  | aPTT | 45.3 seconds | 23.9–35.5 seconds |
|  | D-dimer | 4.53 mg/L FEU | ≤ 0.55 mg/L FEU |
|  | Protein C | 104% | 70–140% |
|  | Protein S | 82% | 58.6–126% |

ALT, alanine aminotransferase; aPTT, activated partial thromboplastin time; AST, aspartate aminotransferase; CRP, C-reactive protein; FEU, fibrinogen-equivalent units; HbA1c, glycated hemoglobin; INR, international normalized ratio; PT, prothrombin time; WBC, white blood cell.
